# Lrp Acts as Both a Positive and Negative Regulator for Type 1 Fimbriae Production in *Salmonella enterica* Serovar Typhimurium

**DOI:** 10.1371/journal.pone.0026896

**Published:** 2011-10-28

**Authors:** Chang-Ho Baek, Ho-Young Kang, Kenneth L. Roland, Roy Curtiss

**Affiliations:** 1 The Biodesign Institute at Arizona State University, Tempe, Arizona, United States of America; 2 School of Life Sciences at Arizona State University, Tempe, Arizona, United States of America; 3 Department of Microbiology, Pusan National University, Pusan, Korea; Indian Institute of Science, India

## Abstract

Leucine-responsive regulatory protein (Lrp) is known to be an indirect activator of type 1 fimbriae synthesis in *Salmonella enterica* serovar Typhimurium via direct regulation of FimZ, a direct positive regulator for type 1 fimbriae production. Using RT-PCR, we have shown previously that *fimA* transcription is dramatically impaired in both *lrp*-deletion (Δ*lrp*) and constitutive-*lrp* expression (*lrp^C^*) mutant strains. In this work, we used chromosomal P_fimA_-*lacZ* fusions and yeast agglutination assays to confirm and extend our previous results. Direct binding of Lrp to P_fimA_ was shown by an electrophoretic mobility shift assay (EMSA) and DNA footprinting assay. Site-directed mutagenesis revealed that the Lrp-binding motifs in P_fimA_ play a role in both activation and repression of type 1 fimbriae production. Overproduction of Lrp also abrogates *fimZ* expression. EMSA data showed that Lrp and FimZ proteins independently bind to P_fimA_ without competitive exclusion. In addition, both Lrp and FimZ binding to P_fimA_ caused a hyper retardation (supershift) of the DNA-protein complex compared to the shift when each protein was present alone. Nutrition-dependent cellular Lrp levels closely correlated with the amount of type 1 fimbriae production. These observations suggest that Lrp plays important roles in type 1 fimbriation by acting as both a positive and negative regulator and its effect depends, at least in part, on the cellular concentration of Lrp in response to the nutritional environment.

## Introduction

Type 1 fimbriae are mannose-sensitive agglutination factors that mediate bacterial adhesion to a broad range of eukaryotic cells by interactions with mannosylated glycoproteins [1]–[3]. Most members of the family *Enterobacteriaceae*, including *Salmonella enterica* serovar Typhimurium, produce type 1 fimbriae that are believed to contribute to pathogenesis by facilitating the initial interaction with host cells [3]–[5]. The *fim* gene cluster, responsible for type 1 fimbriae production, is composed of six structural genes, *fimAICDHF* transcribed as an operon from the *fimA* promoter, three regulatory genes, *fimZ*, *fimY*, and *fimW*, and an arginine tRNA gene, *fimU* [6]. The structural gene products are the major type 1 fimbrial subunit FimA [7], [8], fimblin-like protein FimI [9], periplasmic chaperone FimC [10], outer membrane usher protein FimD [6], minor fimbrial subunit FimH (adhesin) [11], and fimbrial-like protein FimF [6], [11]. The regulatory *fimZ, fimY*, and *fimW* genes are expressed from independent promoters [12]–[14]. The FimZ regulator activates expression of the *fimAICDHF* operon by binding to the *fimA* promoter [15]. In serovar Typhimurium, FimY and FimW act as a transcriptional coactivator and repressor, respectively, through protein-protein interactions with FimZ [12], [14]. However, Saini *et al.* reported that FimY independently activates the *fimA* promoter, and FimW acts as a negative regulator by repressing FimY transcription [16]. The *fimU* gene product arginine tRNA acts as a posttranscriptional regulator by affecting FimY translation [17], [18].

Bacteria are efficient at switching between type 1 fimbriate and non-fimbriate status in response to environmental conditions [3], [19]. The mechanism of phase-variable type 1 fimbriae synthesis has been well characterized in *Escherichia coli* [20]. FimB and FimE recombinases mediate site-specific recombination of the *fimA* promoter region, resulting in alteration of orientations allowing or blocking transcription [20], [21]. Nucleoid-binding global regulators that modulate DNA topology, such as Lrp, integration host factor (IHF), and H-NS affect phase variation and synthesis of type 1 fimbriae in *E. coli* [22]–[27]. In addition, McClain *et al.* suggested that there is an inversion-independent phase variation mechanism [28]. Despite significant homology between the *fim* structural genes, the mechanism by which type 1 fimbriae synthesis is regulated in *S. enterica* serovar Typhimurium differs substantially from that in *E. coli*. The serovar Typhimurium *fimA* promoter does not possess a *cis*-acting regulatory DNA element for reversible inversion-dependent regulation of type 1 fimbriae expression [29]. Moreover, homologs of the *E. coli* FimB and FimE recombinase are not present in serovar Typhimurium [30], [31]. Conversely, no homologs for serovar Typhimurium FimZ, FimY and FimW regulators have been found within the *E. coli fim* gene cluster [32]. In serovar Typhimurium, Lrp is required for type 1 fimbriae production by activating FimZ synthesis [33], whereas in *E. coli*, Lrp is involved in inversion-dependent phase variation [26]. Lrp activates *fimZ* expression by binding directly to the P*_fimZ_* promoter [33]. FimZ is an essential positive regulator for type 1 fimbriae production in serovar Typhimurium [13]. Thus an *lrp*-deletion mutant cannot produce FimZ, and is blocked for type 1 fimbriation [33].

Although, the mechanism for type 1 fimbriae production in bacteria has been extensively studied, no clear mechanism for on-off switching in response to environmental cues has been demonstrated. We have proposed that dynamic change in cellular Lrp levels in response to nutritional state (feast or famine) is important for coordinating virulence traits in *Salmonella* [34]. In this study, we address the effect of Lrp on type 1 fimbriation in *Salmonella*.

## Results

### Lrp acts as both positive and negative regulator for *fimA* expression

**Figure 1:**
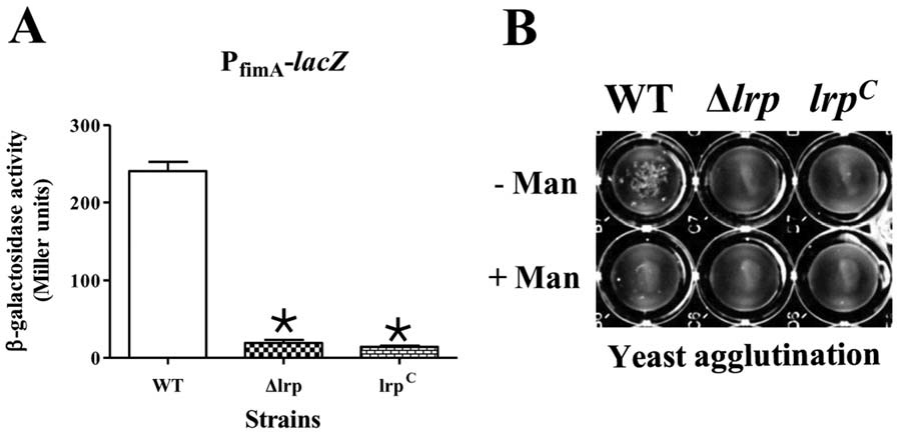
Expression of type 1 fimbrial operon and the associated phenotype in the wild-type (WT, χ3761), Δ*lrp* (χ9411), and *lrp^C^* (χ9448) strains. (A) β-galactosidase assay for the P_fimA_-*lacZ* fusions in each strain is shown. *, *P<*0.05 (B) Mannose-sensitive yeast agglutination assay to assess type 1 fimbriae synthesis. Representative images from several experiments are shown. Bacterial cells were statically grown in LB broth for 24 h at 37°C.

In our previous study using RT-PCR, we observed that an *lrp^C^* (constitutive Lrp expression) mutation abrogated *fimA* transcription [34]. The lack of *fimA* expression in the *lrp^C^* strain was unexpected, since Lrp is known to be an indirect positive regulator for type 1 fimbriae production by enhancing expression of the positive regulator FimZ [33]. To further define the role of Lrp in *fimA* expression, we determined the activity of P_fimA_ using P_fimA_-*lacZ* fusions in wild-type strain χ3761, and isogenic Δ*lrp* and *lrp^C^* mutant strains (Table 1) by measuring the β-galactosidase activity in each of these strains after static, 24 h growth in LB medium at 37°C (Fig. 1A). The lack of β-galactosidase synthesis in the Δ*lrp* and *lrp^C^* mutant backgrounds indicates that transcription from P_fimA_ is not active in the absence of Lrp or when Lrp is overproduced.

**Table 1:** Bacterial strains and plasmids used in this study.

Strains	Description^a^ [parental strain]	Source
***S.*** **Typhimurium**		
**χ3761**	Wild-type strain UK-1, highly virulent for chicks and mice	[52], [53]
**χ9411**	Δ*lrp-13* (*lrp*-deletion mutation) [χ3761]	[34]
**χ9448**	*lrp-1281* (ΔP_lrp_::P_trc_ *lrp*, chromosomal deletion-insertion mutation to drive constitutive expression of Lrp (*lrp^C^*)) [χ3761]	[34]
**χ9449**	Δ*relA198*::*araC* P_BAD_ *lacI* TT Δ*araBAD23 lrp-1281* [χ9509]	[34]
**χ9455**	P_fimA_::pYA4311 (P_fimA_-*lacZ*), Amp^r^ Gm^r^ [χ3761]	This study
**χ9467**	Δ*lrp-13* P_fimA_::pYA4311 (P_fimA_-*lacZ*), Amp^r^ Gm^r^ [χ9411]	This study
**χ11107**	P_fimA413_ (α mutation) [χ3761]	This study
**χ11111**	P_fimA529_ (β mutation) [χ3761]	This study
**χ11115**	P_fimA1225_ (γ mutation) [χ3761]	This study
**χ11153**	*lrp-1281* P_fimA529_ [χ9448]	This study
**χ11263**	*lrp-1281* P_fimA_::pYA4311 (P_fimA_-*lacZ*), Amp^r^ Gm^r^ [χ9448]	This study
**χ11264**	P_fimA413_::pYA4311 (P_fimA413_-*lacZ*), Amp^r^ Gm^r^ [χ11107]	This study
**χ11265**	P_fimA529_::pYA4311 (P_fimA529_-*lacZ*), Amp^r^ Gm^r^ [χ11111]	This study
**χ11266**	P_fimA1225_::pYA4311 (P_fimA1225_-*lacZ*), Amp^r^ Gm^r^ [χ11115]	This study
**χ11267**	*lrp-1281* P_fimA529_::pYA4311 (P_fimA529_-*lacZ*), Amp^r^ Gm^r^ [χ11153]	This study
**χ11377**	P_fimA1325_ (αγ double mutation) [χ11115]	This study
**χ11378**	P_fimA1329_ (αβ double mutation) [χ11107]	This study
**χ11379**	P_fimA2925_ (βγ double mutation) [χ11115]	This study
**χ11380**	P_fimA395_ (αβγ triple mutation) [χ11379]	This study
***E. coli***		
**MGN-617 (χ7213)**	*thr-1 leuB6 fhuA21 lacY1 glnV44 recA1* Δ*asdA4 thi-1* RP4-2-Tc::Mu [λ-*pir*], Km^r^	[54]
**Plasmids**		
**pRE112**	Positive selection suicide vector (R6K *ori*) for gene replacement, Cm^r^	[55]
**pSG3**	a suicide vector (R6K *ori*) for construction of promoter-*lacZ* fusion into chromosome	[49]
**pWSK29**	a low-copy-number cloning vector (pSC101 *ori*), Amp^r^	[47]
**pYA4124**	Derivative of pET SUMO containing His Tag from pET-14b, Km^r^	[34]
**pYA4311**	Derivative of pSG3 for insertion of the P_fimA_-*lacZ* fusion into the chromosome, Amp^r^ Gm^r^	This study
**pYA4758**	Derivative of pRE112 for replacement of the P_fimA_ with P_fimA413_, Cm^r^	This study
**pYA4759**	Derivative of pRE112 for replacement of the P_fimA_ with P_fimA529_, Cm^r^	This study
**pYA4801**	Derivative of pRE112 for replacement of the P_fimA_ with P_fimA1225_, Cm^r^	This study
**pYA4865**	Derivative of pWSK29 harboring a recombinant *fimZ* gene, Amp^r^	This study

a Amp^r^, ampicillin resistance; Gm^r^, gentamicin resistance; Cm^r^, chloramphenicol resistance; Km^r^, kanamycin resistance.

Next, we measured fimbriae production by determining the ability of static cultures of strains χ3761, χ9411 (Δ*lrp*) and χ9448 (*lrp^C^*) to agglutinate yeast cells. Wild-type cells displayed mannose-sensitive agglutination, while both mutant strains were deficient in this phenotype (Fig. 1B), consistent with our observations that no *fimA* transcript was detected in the strains. We further confirmed these results by transmission electron microscopy (TEM). Typical type 1 fimbriae appendages were detected on the cell surface of wild-type strain χ3761, while no type 1 fimbriae were detected on the cell surface of the Δ*lrp* or the *lrp^C^* strains (data not shown). These results demonstrate that deletion of *lrp* or overproduction of Lrp has a strong negative effect on type 1 fimbriae synthesis by directly influencing *fimA* transcription.

### Lrp directly interacts with P_fimA_

**Figure 2:**
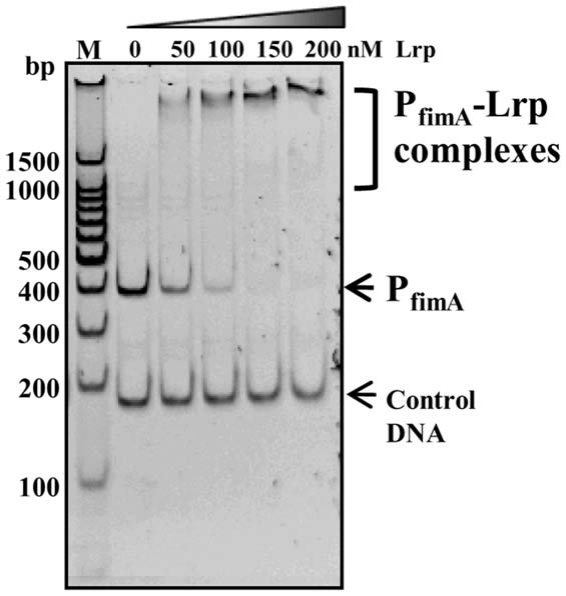
Binding of the purified Lrp to the wild-type P_fimA_. Binding reactions were carried out in various Lrp concentrations: 0, 50, 100, 150, and 200 nM. The 178-bp DNA fragment from pBluescript multi-cloning sites was used as the negative control.

Based on our previously described consensus sequences [34], we detected four putative Lrp-binding motifs in the P_fimA_ region. All four motifs are located upstream of the *fimA* transcription start site (+1) [15]. The DNA motifs 1 [−308 to −301], 2 [−112 to −105], and 3 [−35 to −28] belong to Lrp-binding consensus IV, 5′- GNN(N)TTTT -3′ [34], [35] and DNA motif 4 [−31 to −20] belongs to Lrp-binding consensus III, 5′- HNDWTTATTHND -3′ [where H = not G; W = A or T; D = not C; N = all bases; and (N) = all bases or none] [34]. DNA motif 2 lies just upstream and motifs 3 and 4 lie just downstream of the FimZ binding site [−98 to −47] [33]. These observations led us to postulate that Lrp acts as both an activator and a repressor for type 1 fimbriae expression mediated by differential interactions with Lrp-P_fimA_, depending on cellular concentration of Lrp and on environmental conditions. To address this hypothesis, we tested the direct interaction between Lrp and P_fimA_ using the electrophoretic mobility shift assay (EMSA). Lrp directly interacted with P_fimA_ in a concentration-dependent manner (Fig. 2). These results are in contrast to the study by McFarland *et al* who did not detect an Lrp-P_fimA_ interaction in gel shift assays [33]. We noted differences in the binding buffer used in their study compared to ours. Of particular interest was the fact that their binding buffer included MgCl_2_, while ours did not include divalent cations and, instead included EDTA to chelate any divalent cations present. We performed the EMSA assay using McFarland's binding buffer and, like McFarland *et al*, did not detect binding (data not shown), suggesting that the magnesium concentration may play a role in regulating Lrp binding to P_fimA_.

**Figure 3:**
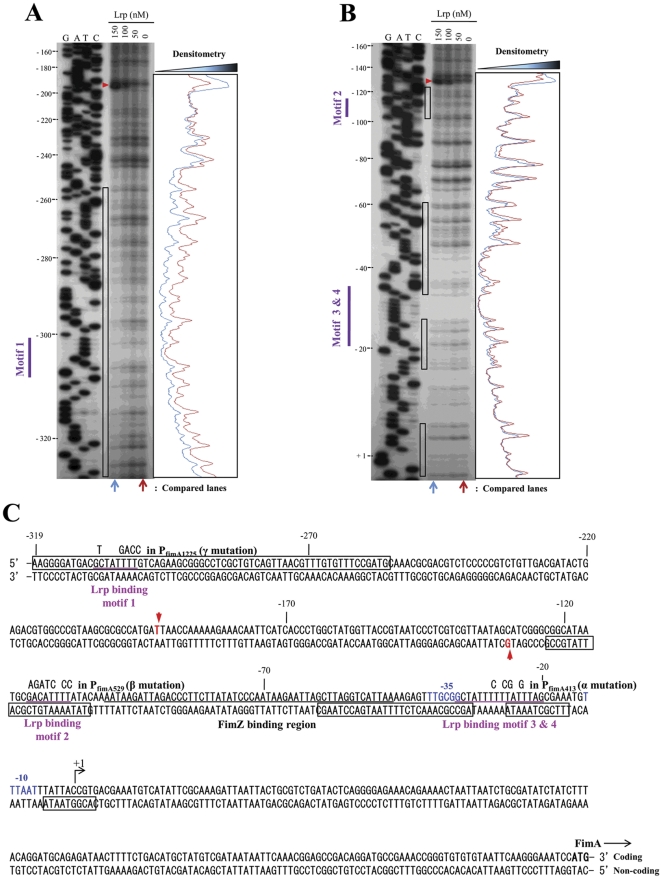
DNase I footprinting of Lrp binding to the P_fimA_ region. Both coding (A) and noncoding (B) strands were subjected to the DNase I protection assay. These strands were ^32^P-labeled at 5′ ends as described in Materials and Methods. Lrp was added at 150, 100, 50, and 0 nM. The DNase I protection products were separated in a sequencing gel next to the corresponding DNA sequencing products (lanes G, A, T, and C). The results from panels A and B are summarized in panel C. The coordinates in the panels A, B, and C are numbered with respect to the *fimA* transcription start site (+1) [15]. The black open boxes indicate DNA bases that were protected from DNase I digestion by Lrp. Hypersensitive bases are indicated with red arrowheads. The putative Lrp-binding motifs are shown as purple bars on the left side of the gels, and are underlined (purple) in panel C. Base changes in the site-directed mutations of the Lrp-binding motifs are shown over the wild-type bases. The FimZ-binding region [15] is also underlined (black). The putative −35 and −10 consensus sequences for RNA polymerase are shown in blue letters. The translation start codon (ATG) for the *fimA* gene is shown in bold letters. Arrows indicate orientation of transcription or translation.

A DNase I footprinting analysis was performed to elucidate in more detail the molecular nature of the Lrp-P_fimA_ interaction with both coding (Fig. 3A) and non-coding (Fig. 3B) strands. A 388-bp DNA probe extending from -334 to +54 with respect to transcriptional start site (+1) was used, which includes the entire P_fimA_ region. The footprint was estimated by densitometry comparing two lanes for 150 nM and 0 nM Lrp. Sites protected from or hypersensitive to DNase I are summarized in Fig. 3C. All four putative Lrp-binding motifs were protected by Lrp (Fig. 3A and B). We observed strong protection of the DNA region (−326 to −257) containing the Lrp-binding motif 1, while protection of the DNA regions (−123 to −102; −59 to −33; and −26 to −6) of the Lrp-binding motifs 2, 3, and 4, respectively, was weaker. The DNA region (−5 to +4) in immediate downstream of the Lrp-binding motif 4 also showed weak protection. The AT-rich overlapping region (−34 to −28) within Lrp-binding motif 3 and 4 was highly resistant to DNase I digestion. The FimZ-binding region partly overlapped with the Lrp-binding region (−59 to −47) (Fig. 3C). This result suggests that both Lrp and FimZ competitively interact with the overlapped motif in P_fimA_. Two super-hypersensitive positions (red arrowheads in Fig. 3A and B), −193 T on coding strand and −130 G on non-coding strand, were detected. None of tested DNA fragments showed non-specific degradation in the absence of DNase I (data not shown).

### Each of the Lrp-binding motifs in P_fimA_ plays a distinct role in regulating type 1 fimbriae production

To dissect the role of Lrp interactions with the promoter region of *fimA*, we constructed P_fimA_ mutations, P_fimA413_, P_fimA529_, and P_fimA1225_, designated α, β, and γ, respectively, in the Lrp-binding motifs by site-directed mutagenesis (Table 1 and Fig. 3C). Our strategy for these mutations was to change multiple bases in each of the Lrp-binding motifs to increase the likelihood of disrupting the Lrp-DNA interaction and to reduce the likelihood of reversion. In addition, the GC content (25% or less) of the Lrp-binding motifs was increased by the changes to achieve a GC content closer to the average for *Salmonella*, approximately 52% [6]. The GC content was raised to 50% by the α and β mutations and to 44% by the γ mutation. None of the changes affected bases known to be part of the RNA polymerase binding site for P_fimA_.

**Figure 4:**
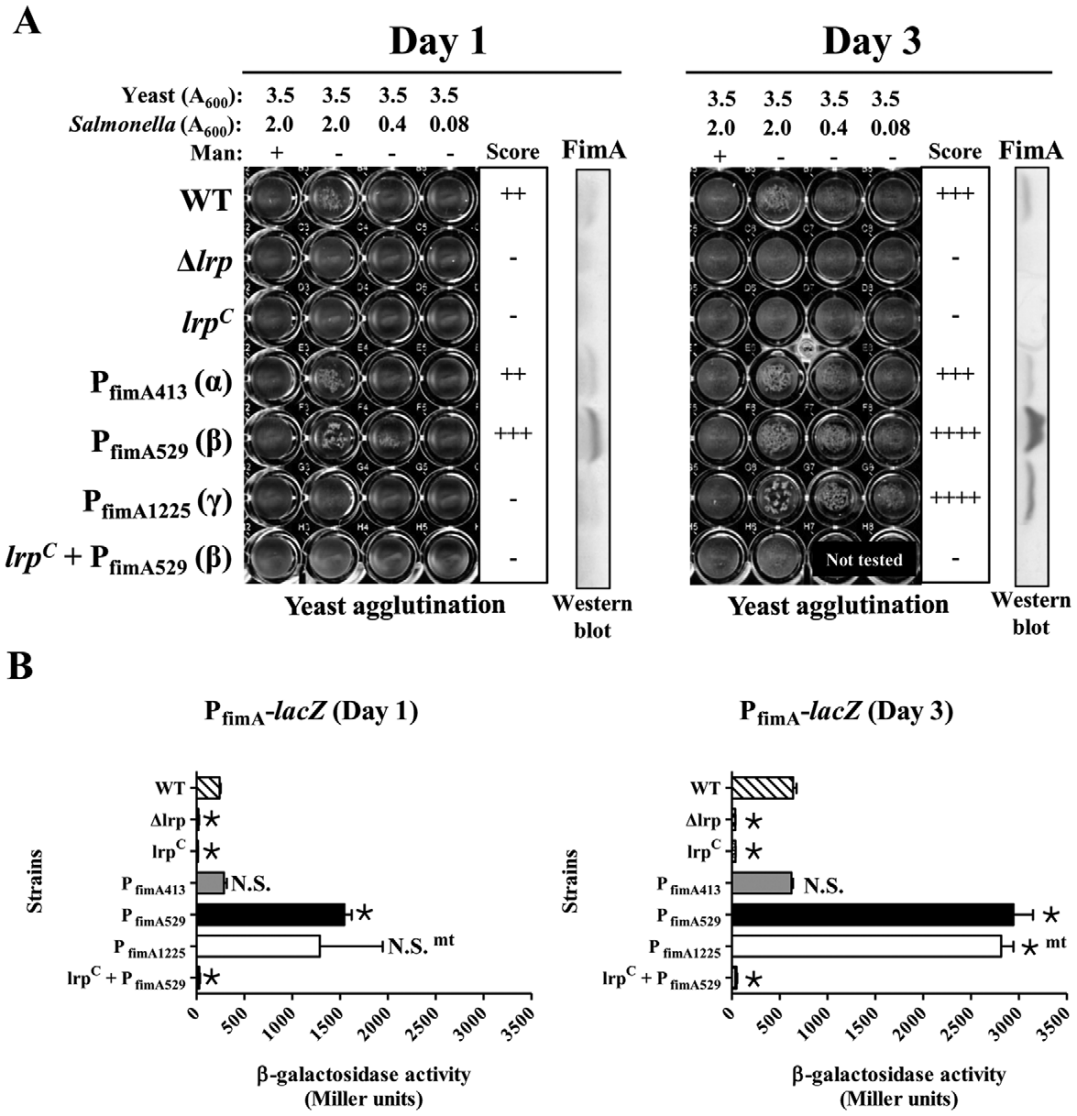
Effect of Lrp-binding motif mutations in P_fimA_ on type 1 fimbriae production and β-galactosidase synthesis directed by P_fimA_ promoter fusions in the indicated *Salmonella* strains. *Salmonella* strains each harboring one of the P_fimA_ mutations were statically grown in LB broth at 37°C for one day (24 h) or 3 days. Mannose-sensitive yeast agglutination assay and western blot analysis were performed to monitor *fim* expression in the single mutants (A). Agglutination was scored as follows: -, none; ++, good; +++, strong; and ++++, very strong. Immunoblots using anti-FimA serum in each panel determine the level of FimA synthesized in each mutant. (B) β-galactosidase assay for a each *lacZ* fusion strain is shown. *, *P<*0.05; N.S., not significant.

To determine the influence of the P_fimA_ mutations on expression, we estimated the levels of mannose-sensitive fimbriae by a yeast agglutination assay, the synthesis of FimA by western blot, and *fimA* expression using chromosomal *fimA*-*lacZ* fusions in strains carrying the promoter mutations (Fig. 4). The P_fimA413_ (α) mutation had no significant effect on yeast agglutination, FimA synthesis or *fimA* transcription when compared to wild-type P_fimA_ [P_fimA_(WT)] (Fig. 4A and B). In contrast, the P_fimA529_ (β) mutation resulted in a substantial increase in FimA synthesis (Fig. 4A) and a simultaneous increase in agglutination even at the lowest cell density used. In contrast, the P_fimA1225_ (γ) mutation resulted in loss of the agglutination phenotype and no detectable FimA synthesis on day 1 of growth (Fig. 4A). These results suggest that Lrp-binding motifs 1 and 2 in the P_fimA_ region are involved in Lrp-mediated activation and repression, respectively, of type 1 fimbrial gene expression. Overall, the results from the lacZ fusion studies mirrored the yeast agglutination and western blot results (Fig. 4A, 4B). The double and triple mutants, P_fimA1325_ (αγ), P_fimA1329_ (αβ), P_fimA2925_ (βγ), and P_fimA395_ (αβγ) displayed wild-type levels of yeast agglutination on both day 1 and day 3 (data not shown).

Interestingly, we observed wide experiment-to-experiment variations in *fimA* expression on day 1, as measured by β-galactosidase synthesis from the P_fimA1225_ (γ)-*lacZ* fusion (Fig. 4B). Yeast agglutination results from the γ mutant (culture at day 1) were also variable among independent experiments (data not shown). Upon plating the γ mutant cultures after three days of growth, we observed heterogeneous colony morphologies, including large and small colonies. Some of the large colonies were purified and retested. They appeared to be highly fimbriated, as they agglutinated yeast strongly after 24 hours of static growth and synthesized FimA (data not shown), suggesting that they had acquired a suppressor mutation. This observation could explain the variable data from the original γ-*lacZ*-fusion mutant culture at day 1 (Fig. 4B). By day 3, we observed strong yeast agglutination (Fig. 4A) and higher levels of β-galactosidase synthesis than wild type (Fig. 4B), consistent with the accumulation of these highly fimbriated, faster-growing spontaneous mutants in the culture (data not shown). To determine whether or not the suppressor mutation was in the *fim* regulatory region, we picked four of these mutants and determined the DNA sequence of the entire promoter region. However, we could not find any additional mutations in the P_fimA1225_ (γ) DNA sequence from the suppressor mutants, indicating that the suppressor mutation is located elsewhere in the chromosome (data not shown).

### Each of the Lrp-binding motifs in P_fimA_ contributes to Lrp-P_fimA_ interaction

**Figure 5:**
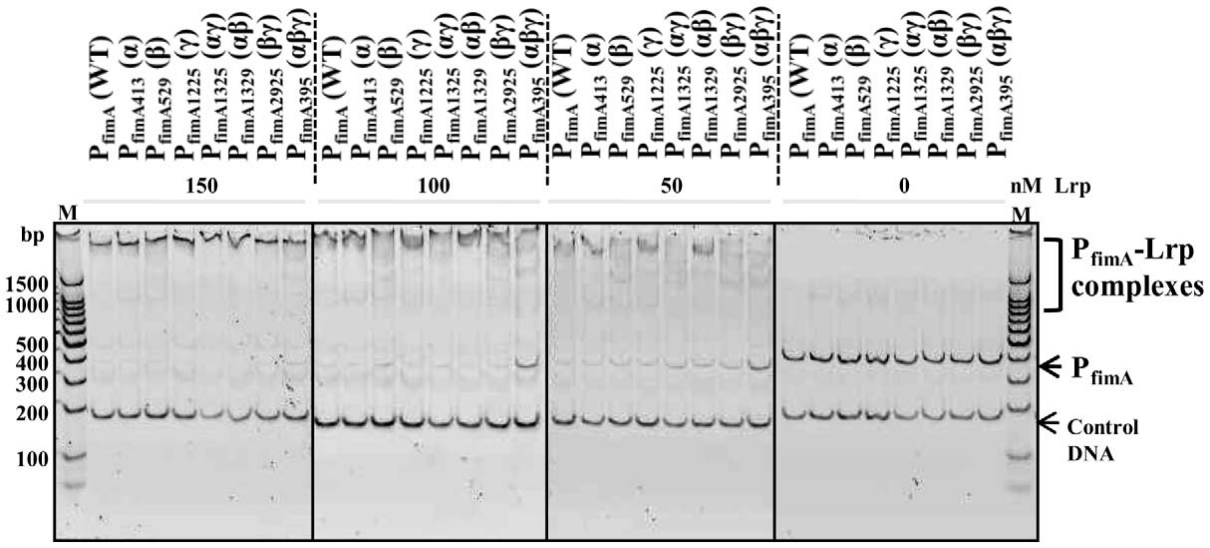
Effects of Lrp-binding motif mutations in P_fimA_ by EMSA. Lrp was added at 150, 100, 50, and 0 nM. Reaction products were separated in a 5% polyacrylamide gel. Data presented are representative of two independent observations.

To address the mechanism behind the Lrp-mediated dual (activation/repression) regulation, interactions between Lrp and each of the mutant *fimA* promoters were evaluated using EMSA. Lrp binding to the the α, β, γ, αγ, αβ, and βγ (single and double) mutant promoters was indistinguishable from binding to P_fimA_(WT) regardless of Lrp concentrations. In contrast, Lrp binding to the αβγ triple mutant promoter was impaired when the Lrp concentration was reduced to 100 nM or 50 nM (Fig. 5). This indicates that each of the Lrp-binding motifs in P_fimA_ contributes to Lrp-P_fimA_ interactions. Leucine had a minor effect on the band pattern, but we observed no significant effect on the binding affinity of Lrp to P_fimA_(WT) (data not shown). In addition, there was no significant effect of leucine on the Lrp-P_fimA_ footprint (data not shown).

### Lrp acts as both positive and negative regulator of *fimZ* expression

**Figure 6:**
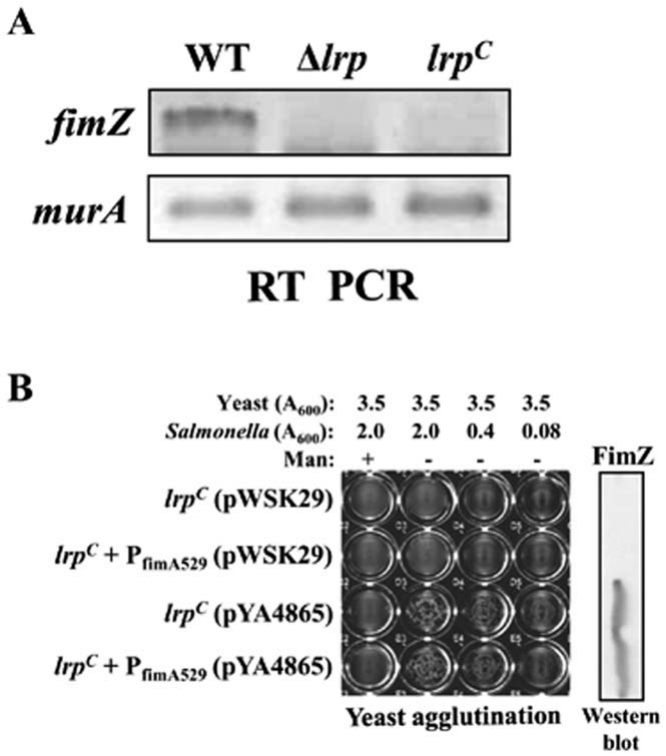
Effect of cellular Lrp levels on FimZ synthesis and P_fimA_-FimZ interaction in *Salmonella*. (A) RT-PCR analysis of *fimZ* transcript in the wild-type (WT, χ3761), Δ*lrp* (χ9411), and *lrp^C^* (χ9448) strains. RT-PCR analysis of *murA* transcript in the strains was used as the control. (B) Mannose-sensitive yeast agglutination assay to assess type 1 fimbriae production and western bolt analysis for the FimZ synthesis in the *lrp^C^* mutants harboring pWSK29 (a low-copy number vector control) or pYA4865 (Lrp-independent *fimZ* expression vector).

The increase in *fimA* expression observed in the β mutant (Fig. 4B) raised the possibility that the binding of Lrp to motif 2, adjacent to the FimZ binding site (Fig. 3C), may affect FimZ binding. If the β mutation precluded Lrp binding, this could allow for greater accessibility of FimZ to its binding site in P_fimA_, thereby accounting for the observed hyper-fimbriation phenotype of the β mutant. To address this possibility, we investigated whether the β mutation could relieve the observed repression of *fimA* in the *lrp^C^* mutant. Therefore, we introduced the β mutation into the *lrp^C^* mutant and evaluated the resulting strain. We found that introduction of the β mutation did not alleviate the Fim^-^ phenotype in *lrp^C^* mutant (Fig. 4). The new strain was essentially identical to the *lrp^C^* mutant carrying the P_fimA_ (WT). It did not produce any detectable FimA (Fig. 4A), did not agglutinate yeast cells, and no *fimA* transcription was detected from the β mutant promoter (Fig. 4B). Because FimZ is a positive activator of *fimA* expression, we assessed *fimZ* expression in the *lrp^C^* mutant by RT-PCR analysis. We found that *fimZ* expression was undetectable in both the Δ*lrp* and *lrp^C^* mutants (Fig. 6A). The results within the Δ*lrp* mutant are consistent with previous observations that Lrp is a positive activator of *fimZ* [33]. The lack of *fimZ* expression in the *lrp^C^* mutant indicates that Lrp can also act as a negative regulator of *fimZ*. In addition, complementation with plasmid-borne (Lrp-independent) *fimZ* can overcome the loss of type 1 fimbriae production in the *lrp^C^* mutant in both wild-type P_fimA_ and P_fimA529_ (β) backgrounds (Fig. 6B). Therefore, it appears that even if Lrp binding is reduced at the Lrp-binding motif 2 when the β mutation is present, no *fimA* is expressed due to repression of *fimZ*. In addition, positive regulation of *fimA* expression by FimZ is dominant over the negative regulation by Lrp when *fimZ* is overexpressed.

### Both Lrp and FimZ independently bind to P_fimA_

**Figure 7:**
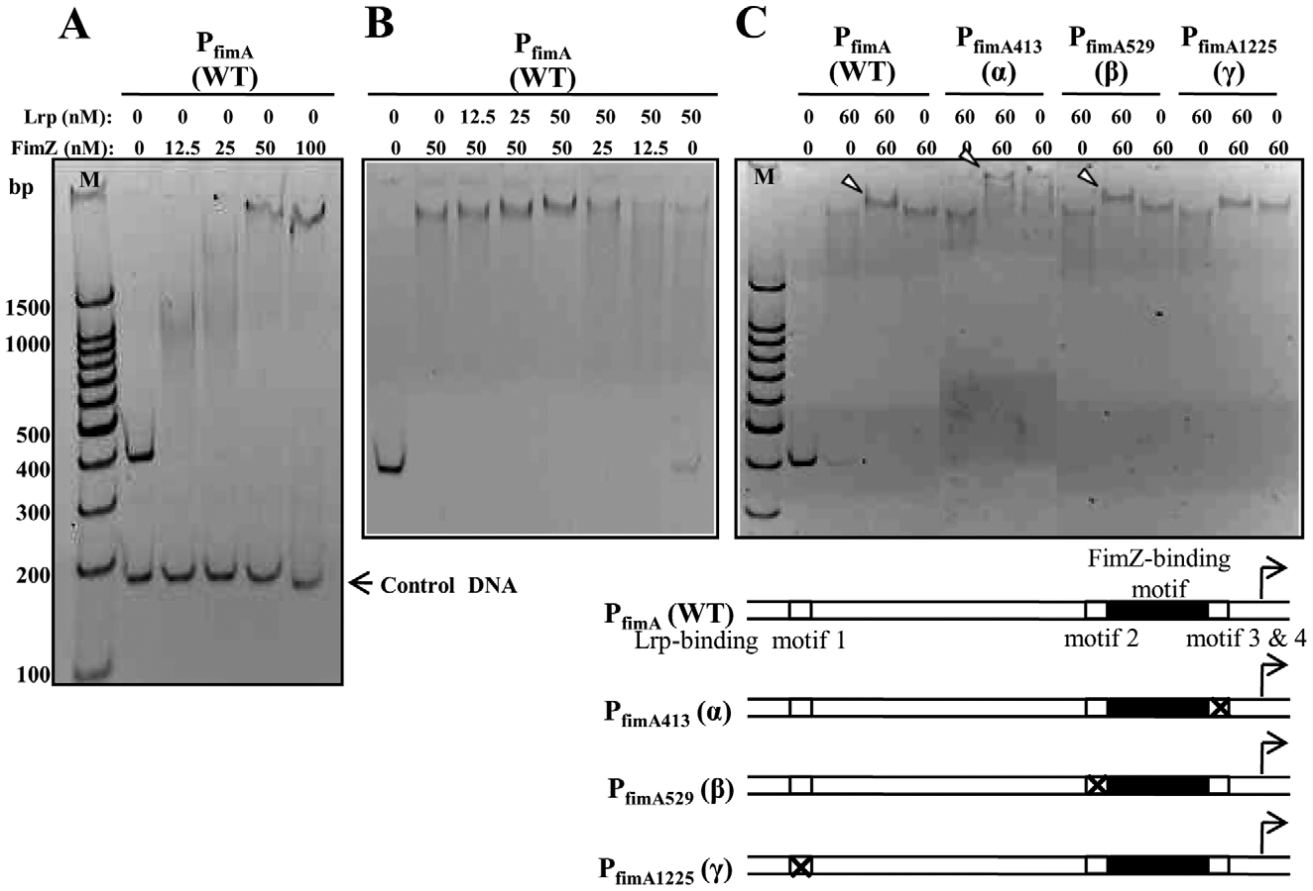
Binding of the purified Lrp and FimZ proteins to P_fimA_. (A) Binding of FimZ to P_fimA_(WT). (B) Binding of Lrp and FimZ to P_fimA_(WT). (C) Binding of Lrp and FimZ to P_fimA_(WT), P_fimA413_ (α), P_fimA529_ (β), or P_fimA1225_ (γ). The white arrowheads indicate super-shifted Lrp-P_fimA_-FimZ complexes. To enhance resolution of the super-shifted DNA-protein complexes, the running time of the polyacrylamide gel in panel C was extended from 1 h (used in panels A and B) to 2 h. Schematic diagrams of the wild-type and mutant *fimA* promoters are shown under the gel.

To gain greater insight into the regulation of *fimA* expression by Lrp and FimZ, we evaluated the binding of Lrp and FimZ to P_fimA_ at several different molar ratios. First, we confirmed that purified FimZ directly interacted with P_fimA_ (Fig. 7A). When the concentration of FimZ was held constant at 50 nM, the intensity of shifted DNA-protein complex was increased by adding Lrp in a concentration dependent manner (Fig. 7B). Similarly, when the Lrp concentration was held constant at 50 nM, the intensity of shifted DNA-protein complex was increased by adding FimZ in a concentration dependent manner (Fig. 7B). In contrast to these conditions, in presence of Lrp (50 nM) or FimZ (12.5 nM) alone, the P_fimA_-Lrp or P_fimA_-FimZ complexes ran as a smear in the gel (Fig. 7A and B). These results indicate that both Lrp and FimZ independently bind to P_fimA_. Although, DNase I footprinting analysis showed that the FimZ-binding motif partly overlapped with the Lrp-binding region (Fig. 3B and C), we could not obtain any evidence for competitive binding of Lrp and FimZ to P_fimA_ using EMSA. In addition, in the presence of both Lrp and FimZ, we observed an increase in the apparent size of the P_fimA_(WT) complex (supershift) compared to the shift when each protein was present alone (Fig. 7C). To identify the Lrp-binding motif(s) responsible for the supershift, we estimated the binding of Lrp and FimZ to each of the mutant promoters, P_fimA413_ (α), P_fimA529_ (β), and P_fimA1225_ (γ). The γ mutation in motif 1 abrogated the supershift of the DNA-protein complex, while the α and β mutations maintained the supershift (Fig. 7C). Remarkably, the α mutation led to a very strong supershift (Fig. 7C). These results indicate that the Lrp-binding motif 1 and the FimZ-binding motif in P_fimA_ allow the supershifting of the P_fimA_ complex by binding of both Lrp and FimZ to P_fimA_.

### The cellular level of Lrp is a key factor for on/off switching of type 1 fimbriae production in serovar Typhimurium

**Figure 8:**
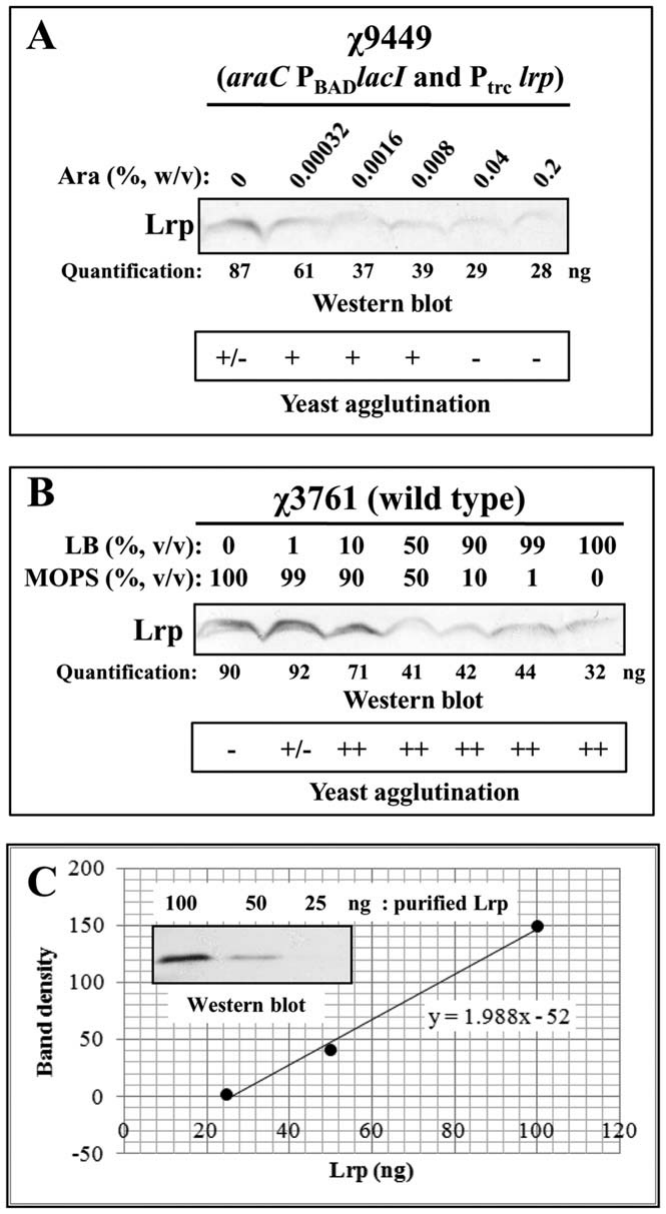
Effect of cellular Lrp levels on type 1 fimbriae production in *S*. Typhimurium. (A) Western blot analysis for Lrp synthesis and mannose-sensitive yeast agglutination assay for the type 1 fimbriation in strain χ9449 grown statically at 37°C for 24 h in LB medium supplemented with various arabinose concentrations. (B) Western blot analysis for Lrp synthesis and mannose-sensitive yeast agglutination assay in the wild-type strain χ3761 grown statically in various combination of MOPS and LB media at 37°C for 24 h. Agglutination was scored as follows: -, no; +/-, very weak; +, weak; and ++, good. (C) Preparation of standard curve for quantification of cellular Lrp levels by western blot analysis of purified Lrp (100, 50, and 25 ng) using anti-Lrp antiserum and densitometry.

To examine Lrp-dependent on/off switching of type 1 fimbriae production, we employed *S*. Typhimurium strain χ9449 harboring an arabinose-dependent Lrp expression system (*araC* P_BAD_ *lacI* and P_trc_ *lrp*) [34]. In the presence of arabinose, *lacI* expression is induced and *lrp* expression, transcribed from the *lacI*-regulated P_trc_ promoter, is repressed. Conversely, in the absence of arabinose, no LacI is produced and Lrp is synthesized (Fig. 8A). In strain χ9449, Lrp synthesis and the ability to agglutinate yeast cells were dependent on the arabinose concentration in LB medium (Fig. 8A).

To evaluate whether the nutrition-dependent cellular Lrp levels are related to on-off switching of type 1 fimbriation, wild-type strain χ3761 was statically grown in MOPS minimal broth, LB broth, and MOPS plus LB (MOPS-LB) broth mixed in several different ratios. Cells from these cultures were harvested and tested for mannose-sensitive yeast agglutination. Cell lysates were analyzed by western blot using anti-Lrp mouse serum. As shown in Fig. 8B, Lrp synthesis was proportional to the nutritional content of the growth medium: with more Lrp produced under poor nutritional conditions and less Lrp produced under rich nutritional conditions. Mannose-sensitive yeast agglutination was dramatically reduced in the *Salmonella* cells grown in MOPS minimal medium, which is the condition generating the highest cellular Lrp level (Fig. 8B). Mannose-sensitive agglutination was partially recovered in the bacterial cells grown in MOPS minimal broth supplemented with 1% (v/v) LB broth (Fig. 8B). The wild-type *Salmonella* completely recovered mannose-sensitive type 1 fimbriation in the MOPS-LB media containing 10% (v/v) LB broth or more (Fig. 8B). These results imply that the nutrition-dependent intracellular Lrp concentration is important for regulating type 1 fimbriation.

To get a better sense of how much Lrp is present in cells, we estimated the number of Lrp molecules per cell when cells were grown in MOPS minimal broth, LB broth, and MOPS plus LB (MOPS-LB) broth mixed in several different ratios by comparing the western blot shown in Fig. 8B with a western blot loaded with known amounts of purified Lrp (Fig. 8C). Based on our densitometry calculations using the standard curve in Fig. 8C, the wild-type *Salmonella* strain χ3761 produced about 6,000 Lrp molecules (3,000 dimers) per cell when grown in MOPS minimal medium. This is the same number of Lrp molecules calculated for *E. coli* cells grown in a glucose-based minimal medium [36]. The wild-type *Salmonella* strain χ3761 produced about 2,000 Lrp molecules (1,000 dimers) per cell in LB medium, whereas the *lrp^C^* mutant produced about 12,000 Lrp molecules (6,000 dimers) per cell grown in LB medium (data not shown). In MOPS minimal medium supplemented with 10% (v/v) LB, χ3761 produced approximately 4,700 Lrp molecules (2,350 dimers) per cell. In addition, the regulated *lrp*-expression mutant χ9449 produced about 900 Lrp dimers per cell when grown in LB medium supplemented with 0.04% or 0.2% arabinose, and did not produce type 1 fimbriae. These results indicate that *S*. Typhimurium is able to produce type 1 fimbriae at a range of cellular Lrp concentrations (from 1,000 to 2,400 Lrp dimers per cell). In MOPS minimal medium supplemented with 1% (v/v) LB, χ3761 produced some type 1 fimbriae, as judged by the mannose-sensitive yeast agglutination assay, even though this strain produced 3,000 Lrp dimers per cell, more than the 2,400 dimers per cell predicted to be the maximum number that would permit *fimA* transcription, based on growth in MOPS. This result suggests that nutritional signals in LB broth may partially relieve the Lrp-mediated repression of type 1 fimbriation at high cellular Lrp concentration. Identification of the nutritional signals in LB medium remains to be addressed.

## Discussion

Lrp is required for synthesis of type 1 fimbriae [33]. Previous studies have shown that FimZ is required for *fimA* expression and Lrp is required for *fimZ* expression [33]. Lrp binds to the *fimZ* promoter region and can thereby enhance *fimZ* expression [33]. FimZ binds to P_fimA_ and activates *fimA* expression. Our previous report showed that while Δ*lrp* mutants did not express *fimA* as expected, neither did *lrp^C^* mutants [34]. To address the basis for these apparently contradictory phenomena, we investigated the role of Lrp in regulating type 1 fimbriae synthesis in more detail. Using both genetic and molecular approaches, we found that high cellular levels of Lrp repressed *fimA* expression, with a concomitant loss of the type 1 fimbriae-associated mannose-sensitive agglutination phenotype (Fig. 4). Under these conditions, production of FimZ is also abrogated (Fig. 6). Site directed mutagenesis of putative Lrp-binding sites in the *fimA* promoter indicated that binding of Lrp to the *fimA* promoter is necessary for both activation and repression of type 1 fimbriae expression (Fig. 4). The γ mutation in the Lrp-binding motif 1 (Fig. 3B) abolished type 1 fimbriae synthesis, as judged by *fimA* expression and the yeast agglutination phenotype (Fig. 4). These results suggest that Lrp-binding motif 1 in P_fimA_ may play a crucial role in Lrp-mediated activation of type 1 production. This notion is supported by the observation that the γ mutation eliminates the formation of a supershifted band in the EMSA assay (Fig. 7C), indicating that Lrp binds to motif 1. In addition, the supershifting of this DNA-protein complex suggests that both Lrp and FimZ binding to P_fimA_ lead to a change in DNA topology, since Lrp changes DNA topology by DNA-protein and protein-protein interactions [37]. This DNA topology change may contribute to activation of type 1 fimbriae production under normal growth conditions in cells (neither the absence nor overproduction of the regulators Lrp or FimZ). The precise mechanism driving the activation of *fimA* transcription remains to be elucidated.

The β mutation in Lrp-binding motif 2 (Fig. 3B) enhanced *fimA* transcription and FimA synthesis (Fig. 4), indicating that the Lrp-binding motif 2 in P_fimA_ is important for repression of type 1 fimbriae production. Based on our results showing that Lrp binds to motif 2 (Fig. 3B), we infer that repression via motif 2 is Lrp-mediated. Introduction of the *lrp^C^* mutation into the β mutant (P_fimA529_) repressed *fimA* expression and eliminated any detectable yeast agglutination (Fig. 6B). This result is most easily explained by the lack of *fimZ* expression in these cells (Fig. 6A), as the synthesis of type 1 fimbriae is restored by overexpression of *fimZ* (Fig. 6B). FimZ-dependent P_fimA_ activation dominates the Lrp effect on P_fimA_ when *fimZ* is overexpressed (Fig. 6) and in fact, does not require Lrp when expressed from a multicopy plasmid [33]. In contrast to the effects observed in the β and γ mutants, the α mutation in Lrp-binding motif 3 and 4 had no effect on agglutination or on transcription of the P_fimA_::*lacZ* fusion (Fig. 4). The DNase I footprinting analysis showed that the protection region at Lrp-binding motif 3 and 4 partly overlapped with the FimZ-binding motif (Fig. 3). This result suggests that Lrp and FimZ compete for binding to P_fimA_ at the overlapping sites. However, the EMSA results indicated that Lrp and FimZ independently bind to P_fimA_ without competitive exclusion.

Many Lrp-regulated genes include multiple Lrp-binding motifs in their promoter region. Cooperative binding of Lrp to these motifs is an important factor for Lrp-mediated gene regulation [38]. Cooperative interactions between Lrp and other nucleoid-binding proteins such as H-NS are thought to repress transcription of some genes [39]. While Lrp has been shown to act as a positive or negative regulator for each of the genes in the Lrp regulon, no systematic study of the mechanism has been undertaken. Although it is unusual for Lrp to be both a positive and a negative regulator in the same operon, this type of dual regulation has been reported for the *papBA* operon [40]. In that case, Lrp interacts with H-NS for repression and PapI for activation.

The P_fimA_ region of *S*. Typhimurium includes four Lrp-binding motifs, 1, 2, 3, and 4 (Fig. 3C). Interestingly, three motifs are located immediately upstream (motif 2) and downstream (3 and 4) of the FimZ-binding *cis* element in P_fimA_ [33]. Motif 1 is located far upstream (−308 to −301) from the *fimA* transcription start site [15]. Moreover, two potential high-affinity H-NS binding sites [41], 5′- AAAATAAGA -3′ (−100 to −92) and 5′- ATTAAAAGA -3′ (−51 to −43), are located immediate downstream of Lrp-binding motif 2 and upstream of Lrp-binding motif 3, respectively, and overlap with the FimZ-binding site. This observation suggests that Lrp binding to Lrp-binding motifs 2 and 3 may facilitate binding of the silencing protein H-NS. Site-directed mutagenesis revealed that motifs 1 (distal locus for γ mutation) and 2 (proximal locus for β mutation) are important determinants for activation and repression, respectively, of type 1 fimbriae production (Fig. 4). This result is consistent with previous reports that Lrp acts as a repressor when bound to motifs closer to or within the promoter and as an activator when bound to motifs further upstream [35], [42]. Similarly, we also found seven Lrp-binding motifs in the P_fimZ_ region (603-bp, between *fimY* stop codon and *fimZ* start codon) (data not shown). Two motifs belong to Lrp-binding consensus III (5′- HNDWTTATTHND -3′) and five motifs belong to Lrp-binding consensus IV (5′- GNN(N)TTTT -3′) [34], [35]. Furthermore, three of the seven motifs in P_fimZ_ are strong Lrp-binding DNA sequences as identified by DNA footprint analysis in a previous study [33]. One Lrp-binding motif in P_fimZ_ is also located far upstream (−353 to −346) from the *fimZ* transcription start site [33] similar to motif 1 in P_fimA_ (Fig. 3C). A specific feature of the P_fimZ_ region is that two Lrp-binding motifs are located between the transcription start site and the start codon of *fimZ* gene. Although the double and triple mutations, P_fimA1325_ (αγ), P_fimA1329_ (αβ), P_fimA2925_ (βγ), and P_fimA395_ (αβγ) still can interact with Lrp, all of the multiple mutants produced wild-type levels of yeast agglutination on both day 1 and day 3 (data not shown). These results suggest that for Lrp to exert its regulatory effect, it must bind to at least two Lrp-binding motifs in P_fimA_. In the absence of cooperative binding, as is the case in the double or triple mutants, P_fimA_ expression would not be under direct Lrp control. However, transcription from these mutant promoters is still sensitive to regulation by FimZ. Thus these promoters can be activated by FimZ and produce wild-type levels of type 1 fimbriae when grown in LB broth. In addition, we believe that the DNA motif 3 and 4 (α mutation position) can facilitate cooperative binding of Lrp to the DNA motif 1 and motif 2, even if α mutation itself does not have any effect on type 1 fimbriae production. Therefore, the effects of β and γ mutations can be suppressed by adding α mutation. These observations indicate that the cooperative binding of Lrp to multiple Lrp-binding motifs in P_fimA_ is important for Lrp-mediated regulation of type 1 fimbriae production. Based on results from site-directed mutagenesis of the multiple Lrp-binding motifs in P_fimA_, we assume that the organization (proximity, number, and orientation) of Lrp-binding motifs and their cooperative interaction with Lrp play a crucial role for on/off switching of the *fimZ* gene expression.

Saini *et al*. suggested that inhibition of *fim* gene expression occurs through the direct repression of P_fimY_ by FimW, resulting in prevention of FimY-mediated *fimZ* activation [16]. FimY is also a transcriptional activator for *fimA*, *fimW*, and itself [14], [16]. However, the positive and negative feedback loops are not sufficient to explain the regulation of type 1 fimbriae synthesis, since type 1 fimbriae synthesis under inducing conditions is continuous or rheostat-like rather than an autocatalytic or switch-like response [16]. These observations suggest that expression of type 1 fimbriae in *Salmonella* cells is a collective and continuous event in response to environmental milieu. These phenomena could be well explained if we assume that Lrp can act as both positive and negative regulators for type 1 fimbriae production depending on intracellular levels of Lrp, which are closely related with the nutritional environment [34], [43]. In addition, mannose-sensitive yeast agglutination correlated with nutrition-dependent cellular Lrp levels (Figs. 8A and B). Based on the Lrp titration results from the western blot analyses in Fig. 7B, we conclude that mannose-sensitive type 1 fimbriation in *Salmonella* is inhibited by Lrp at a concentration of 3,000 or more Lrp dimers per cell (Fig. 8B) under nutrient-poor conditions. However, mannose-sensitive yeast agglutination was observed in *Salmonella* producing about 2,400 or fewer Lrp dimers per cell in nutrient-rich environments (Fig. 8B). We estimated that the *lrp^C^* mutant χ9448 produces at least 6,000 Lrp dimers per cell grown in LB medium. This cellular Lrp concentration is enough to inhibit type 1 fimbriae production, even when the *Salmonella* cells are grown in LB broth, a rich medium. Therefore, our results indicate that a narrow range of Lrp concentrations governs activation of *fimA* transcription and production of type 1 fimbriae. Too much or too little Lrp results in no type 1 fimbriae production, allowing the cell to tightly regulate production of these complex extracellular structures in response to the nutritional environment.

**Figure 9:**
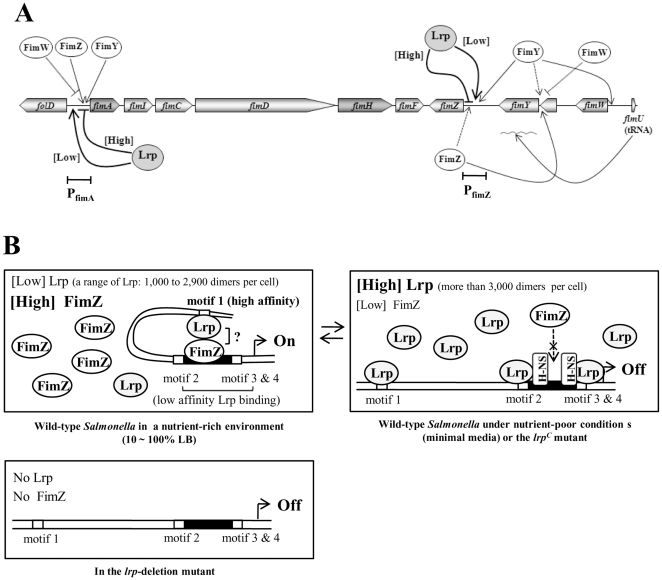
Model for Lrp and FimZ-mediated regulation of type 1 fimbriae production in *Salmonella*. (A) Summary of the regulatory circuit for type 1 fimbriae production. Arrowed and blunted lines indicate activation and repression, respectively. (B) Molecular model for Lrp and FimZ-mediated regulation of type 1 fimbriae production. Dotted arrows, access to binding sites; ×, competitive exclusion of competitor binding; and arrowed flag indicates the transcription start site [15]. [High], at high concentration of Lrp or FimZ; and [Low], at low concentration of Lrp or FimZ.

Based on these data, we propose here a revised model for the *fim* gene regulatory circuit in *S.* Typhimurium (Fig. 9A). In our model, Lrp modulates expression of *fimA* and *fimZ*, either positively or negatively, depending on growth conditions and the amount of Lrp present. When Lrp is present in excess (more than 3,000 dimers per cell), as is the case in the *lrp^C^* mutant or during growth in nutrition-poor media, no *fimZ* is expressed, and Lrp binds to all four motifs 1, 2, 3, and 4 in P_fimA_ resulting in complete repression of *fimA* (Fig. 9B). We assume that the binding of Lrp to motifs 2 and 3 may allow binding of the silencing factor H-NS to the high affinity H-NS-binding motifs in the FimZ-binding region, and competitively exclude FimZ binding to the P_fimA_. This feature is similar to the collaborative competition mechanism in eukaryotic gene regulatory regions typically encompassing multiple DNA target sites for two or more regulatory proteins within a space of a few hundred base pairs or less [44]. At a lower range of Lrp concentrations (about 1,000∼2400 dimers per cell), the levels of FimZ are high, such as occurs when cells are grown in nutrient-rich conditions (e.g. LB broth), FimZ is better able to occupy its activation site in P_fimA_, presumably due to the fact the affinity of Lrp to motifs 2, 3, and 4 is relatively weak (Fig. 3B) and there is an abundance of FimZ. Due to the requirement of motif 1 for FimA and type 1 fimbriae synthesis (Fig 4), we infer an interaction between Lrp and FimZ under these conditions that enhance FimZ-mediated activation of *fimA* expression by changing the regional DNA secondary structure. Finally, in the Δ*lrp* strain, neither *fimZ* nor *fimA* are expressed. We conclude that Lrp is a key regulator to direct on-off switching of type 1 fimbriae production by the concentration-dependent dual regulation in *S*. Typhimurium in contrast to the recombination-mediated phase-variable type 1 fimbriation in *E. coli*.

## Materials and Methods

### Bacterial strains, plasmids, culture conditions, and reagents

Bacterial strains and plasmids used in this study are listed in Table 1. *S*. Typhimurium and *E. coli* strains were routinely grown in LB broth [45]. For analysis of type 1 fimbriae production, *S*. Typhimurium strains were grown statically in MOPS minimal broth [46] or LB broth at 37°C for 24 h or 3 days. Diaminopimelic acid (DAP, 50 µg/ml) was added to LB medium for growing Δ*asd* mutant strains. Antibiotics were used as needed at the following concentrations: ampicillin, 100 µg/ml; chloramphenicol, 20 µg/ml; gentamicin, 20 µg/ml; kanamycin, 50 µg/ml; and tetracycline, 10 µg/ml. All antibiotics and chemicals were purchased from Sigma Chemical Company (St. Louis, MO) or Fisher Scientific Inc (Pittsburgh, PA).

### DNA manipulations

The primers used in this study are listed in Table 2. Plasmid DNA was isolated by using QIAprep Spin Miniprep Kit (QIAGEN, Valencia, CA). Restriction enzymes and DNA-modifying enzymes were used as recommended by the manufacturers (Promega, Madison, WI or New England Biolabs, Ipswich, MA).

**Table 2:** Primers were used in this study.

Name	Sequence (5′ to 3′)	Related product
**RCB-24**	GACCTCTACTATTGCGAG	*fimA*
**RCB-25**	TCAACCAGCGACTGCTTC	*fimA*
**RCB-28**	CCGCGCTAGCGCCGCGCGCGAGCCGGAAATTGTC	*murA*
**RCB-29**	CGCAAGCTTTTCGCCTTTCACGCGTTCAATATTC	*murA*
**RCB-42**	ACTAAAGGGAACAAAAGC	MCS-pBS
**RCB-43**	GTAAAACGACGGCCAGTG	MCS-pBS
**RCB-44**	CGTGGGCCCTCGTCGTTAATAG	P_fimA_-*lacZ*
**RCB-45**	TTAGGATCCATGGATTTCCCTTGA	P_fimA_-*lacZ*
**RCB-46**	CTATTCTCGAGTTAGCGAAATGTTTAATTTATTAC	P_fimA413_
**RCB-47**	TAACTCGAGAATAGCCGCAAACTCTTTTAATG	P_fimA413_
**RCB-48**	TGCAGATCTCCATACAAAATAAGATTAGACCCTTC	P_fimA529_
**RCB-49**	TATGGAGATCTGCATTATGCCGCCCGATG	P_fimA529_
**RCB-50**	GACTCTAGACCGTCAGAAGCGGGCCTCGCTGTC	P_fimA1225_
**RCB-51**	GACGGTCTAGAGTCATCCCCT TTGACTTG	P_fimA1225_
**RCB-52**	ATACCATGGGCAGCAGCCATCATCATCATCATCACAGCAGCGGCATGAAACCTGCATCTG	*fimZ* (RT)
**RCB-53**	CGCGGATCCGACCTTCCTGATCAATTAC	*fimZ* (RT)
**RCB-54**	TGTGGATCCAAGTCAAAGGGGATGAC	P_fimA_ (EMSA)
**RCB-55**	CCTGAGTATCAGACGCAG	P_fimA_ (EMSA)
**RCB-56**	ATCGGGCCCGATACGCTCCAGCAC	P_fimA_
**RCB-57**	TTTGAGCTCGGCTTCAACGGTGAAGA	P_fimA_

To produce a His-tagged FimZ protein, a DNA fragment containing the entire *fimZ* ORF was amplified from strain χ3761 by PCR using primers, RCB-52 and RCB-53 (Table 2). This PCR product was digested with NcoI/BamHI and ligated into expression vector pYA4124 digested with the same enzymes [34]. The recombinant *fimZ* gene was excised from the resulting plasmid using XbaI/BamHI and cloned into a low-copy number plasmid pWSK29 [47] using the same enzyme sites, to create pYA4865.

### Purification of FimZ

χ9448 (pYA4865) (Table 1) was used for synthesis of the His-tagged FimZ fusion protein. Cells were grown to an early stationary phase (optical density at 600 nm [OD_600_] of 1.2) in LB medium at 37°C and harvested by centrifugation at 3,300 × g for 15 min at 4^o^C. The His-tagged FimZ fusion protein was purified by using a nickel affinity gel system, Ni Sepharose 6 Fast Flow (Amersham Bioscience).

### Construction of the P_fimA413_, P_fimA529_, and P_fimA1225_ mutations

The *fimA* promoter (P_fimA_) region was amplified from χ3761 by PCR using primers RCB-56 and RCB-57 (Table 2). The resulting PCR product was digested with ApaI/SacI and ligated with ApaI/SacI-digested pBluescript SK-. The resulting plasmid was used as a template DNA for site directed mutagenesis of the Lrp-binding motifs in P_fimA_ by inverse PCR using primers RCB-46 and RCB-47 for P_fimA413_, RCB-48 and RCB-49 for P_fimA529_ and RCB-50 and RCB-51 for P_fimA1225_ (Table 2). The inverse PCR products were digested with XhoI, BglII, or XbaI, and self-ligated, to create plasmids carrying P_fimA413_, P_fimA529_, and P_fimA1225_ mutations, respectively. The P_fimA413_, P_fimA529_, and P_fimA1225_ DNA fragments were excised using KpnI/SacI restriction enzymes, and cloned into the same restriction enzyme sites of suicide vector pRE112, to create pYA4758, pYA4759, and pYA4801, respectively. These suicide plasmids were introduced into serovar Typhimurium strains by conjugation to construct the P_fimA_ mutants, P_fimA413_, P_fimA529_, and P_fimA1225_, by allelic exchange as previously described [48]. Each of the P_fimA413_ and P_fimA529_ mutations were added to the P_fimA1225_ mutant to generate double mutants P_fimA1325_ and P_fimA2925_, respectively. The P_fimA529_ mutation was added to the P_fimA413_ mutant to generate a double mutant P_fimA1329_. The P_fimA413_ mutation was added to the P_fimA2925_ double mutant to generate a triple mutant P_fimA395_.

### Construction of *lacZ* fusions

A 357-bp DNA fragment containing the *fimA* promoter region was amplified from χ3761 by PCR using primers RCB-44 and RCB-45 (Table 2). The PCR product was digested with ApaI and BamHI, and was cloned into the unique ApaI/BamHI sites of *lacZ*-fusion suicide vector pSG3 [49]. The resulting plasmid pYA4311 was introduced by conjugation into various *Salmonella* strains to obtain P_fimA_-*lacZ* fusions by a single crossover event as previously described [49].

### Yeast agglutination assay

Bacteria were grown statically in various media at 37°C for 24 h and/or 3 days. Bacterial cells were harvested by centrifugation at 5,000× g for 5 min at room temperature. The cell pellet was gently resuspended in phosphate-buffered saline (PBS) and serially diluted in the same buffer to adjust the optical density at 600 nm [OD_600_] to 4.0, 0.8, or 0.16. Yeast cells were washed twice with PBS and diluted to an OD_600_ of 7.0. Agglutination assays were carried out in 96 well microtiter plates by incubating 25 µl of bacterial cell suspension with the same volume of yeast cells in PBS at room temperature for 10 min with gentle orbital shaking. Mannose sensitivity was demonstrated by the absence of agglutination when the assay was performed in the presence of 2% (wt/vol) mannose.

### β-Galactosidase assay

Bacterial cells were statically grown at 37°C for 24 h. β-Galactosidase activity was measured as previously described [50]. Means ± standard errors were calculated from four independent assays.

### Reverse transcription (RT)-PCR analysis

A 0.5 ml aliquot of bacterial culture was mixed with 1 ml of RNAprotect Bacteria Reagent (QIAGEN, Valencia, CA) and incubated for 5 min at room temperature. Cells were harvested by centrifugation at 8,000 × g for 5 min at room temperature. Total RNA from the cell pellet was isolated using RNeasy Mini Kit (QIAGEN). The RNA samples were treated with extra DNase I to avoid any DNA contamination, and repurified using the column in the kit. The DNA-free RNA samples were confirmed by PCR. A 200 ng sample of total RNA was used for semi-quantitative RT-PCR with the OneStep RT-PCR kit (QIAGEN). RT was performed for 30 min at 50°C, followed by heat inactivation of the reverse transcriptase for 15 min at 95°C. PCR amplification was performed in the same tube with the following cycling conditions: 25 cycles with 30 s at 95°C for template denaturation, 30 s at 55°C for primer annealing, and 1 min at 72 °C for primer extension. The primers for RT-PCR (expected sizes of PCR products) were as follows: RCB-24 and RCB-25 for *fimA* (427 bp) [34]; RCB-52 and RCB-53 for *fimZ* (757 bp); RCB-28 and RCB-29 for *murA* (725 bp) [34] (Table 2). PCR products were separated in a 1.0% agarose gel, stained with ethidium bromide, and visualized on a UV transilluminator.

### Electrophoretic Mobility Shift Assay (EMSA)

The wild-type P_fimA_, P_fimA413_, P_fimA529_, P_fimA1225_, P_fimA1325_, P_fimA1329_, P_fimA2925_, and P_fimA395_ DNA fragments were amplified from wild-type and mutant strains by PCR using primers RCB-54 and RCB-55 (Table 2). The multi-cloning site of pBluescript SK- (MCS-pBS, 178 bp) was amplified by PCR using primers, RCB-42 and RCB-43 (Table 2) and used as non-specific control DNA [34]. These DNA fragments were tested for interaction with Lrp protein by EMSA as previously described [34]. The reactions subjected to electrophoretic separation in a 5% polyacrylamide gel in 1× Tris-borate-EDTA (TBE) (100 V for 1 h) or 1 × Tris-taurine-EDTA buffer (TTE) (75 V for 1 h). The gel was stained with 1 × SYBR Gold (Invitrogen), and DNA bands were visualized on a UV transilluminator.

For the competitive binding assay, the P_fimA_ DNA fragment was mixed with different molar ratios of Lrp and FimZ in the DNA binding buffer [15] with minor modification: 5 mM Tris-HCl pH 7.5, 25 mM KCl, 0.5 mM EDTA, 0.5 mM dithiothreitol, BSA (5 µg/ml), and 20% glycerol. The reaction mixture was incubated at room temperature for 15 min, and was subjected to electrophoresis in 1 × TTE buffer as described above.

### DNase I footprinting

RCB-54 and RCB-55 primers were labeled with [γ-^32^P]ATP (PerkinElmer) using DNA 5′ end-labeling system (Promega) for non-coding and coding strands, respectively. The DNA probes were amplified by PCR using primer sets, [γ-^32^P]ATP-labeled RCB-54 and unlabeled RCB-55 primers or vice versa. One pmol of the labeled probe was used for DNase I foot printing analysis. Lrp-DNA probe binding reactions were identical to the conditions for EMSA excepting addition of poly(dI-dC) at 10 µg ml^−1^ (Sigma) instead of MCS-pBS control DNA. One µl volume of DNase I (0.1 unit, Ambion) in 1 × DNase I buffer was added to the reaction and incubated at room temperature for 6 min. Five µl volume of stop buffer (95% formamide, 20 mM EDTA, 0.05% bromophenol blue, and 0.05% xylene cyanol) was added to the reaction mixture and incubated at 75°C for 10 min. The [γ-32P]ATP-labeled RCB-54 and RCB-55 primers were also used for DNA sequencing using Sequi_Therm EXCEL II DNA sequencing Kit (EPICENTRE Biotechnologies). The products of the DNase I footprinting and the DNA sequencing reactions were resolved by electrophoresis through a denaturing 7% polyacrylamide-7M urea gel in 1 × Tris-taurine-EDTA buffer. The gel was subjected to autoradiography.

### Western blot analysis

Protein bands from a 12% SDS polyacrylamide gel were transferred to a nitrocellulose membrane. Western blot analysis was performed as previously described [51]. Blots were probed with rabbit anti-FimA, anti-Lrp, or anti-His6 monoclonal mouse immunoglobulin G (IgG, Invitrogen). Alkaline phosphatase-conjugated goat anti-rabbit IgG or goat anti-mouse IgG (Sigma) was the secondary antibody, as appropriate.

### Estimation of the number of Lrp molecules per cell

The number of *Salmonella* cells per ml was estimated by turbidity and CFU. The Lrp concentration in the crude bacterial cell extract was determined by western blotting next to dilutions of purified Lrp using an anti-Lrp mouse antiserum [34]. We calculated the number of Lrp molecules per ml based on the western blots and then divided the estimated number of Lrp molecules per ml by the number of cells per ml to obtain the number of Lrp molecules per cell.

### Densitometry

The relative band intensities were obtained by using a computational densitometry program Quantity One (Bio-Rad).

### Statistical Analysis

Statistical analysis was performed using t test (GraphPad). Results are presented as the mean and SEM. A *P* value<0.05 was considered statistically significant.
